# 159. Epidemiology of Tuberculosis and HIV within United States immigration detention centers, 2019 through 2023

**DOI:** 10.1093/ofid/ofaf695.054

**Published:** 2026-01-11

**Authors:** Ribhav Gupta, Dean L Winslow, Ronit Gupta, Sten Vermund

**Affiliations:** Stanford University, Stanford, CA; Stanford University, Stanford, CA; Harvard University, Boston, Massachusetts; University of South Florida, Tampa, Florida

## Abstract

**Background:**

Migrants detained by U.S. Immigration and Customs Enforcement (ICE) are likely at higher baseline risk of Tuberculosis (TB) and HIV. Per ICE guidelines, TB screening is mandated on intake with no routine HIV testing protocols.

This study explores TB and HIV epidemiology across ICE facilities from 2019-2023.

Three month sliding average of monthly diagnosed cases over time from 2019 through 2023.

**Figure:**
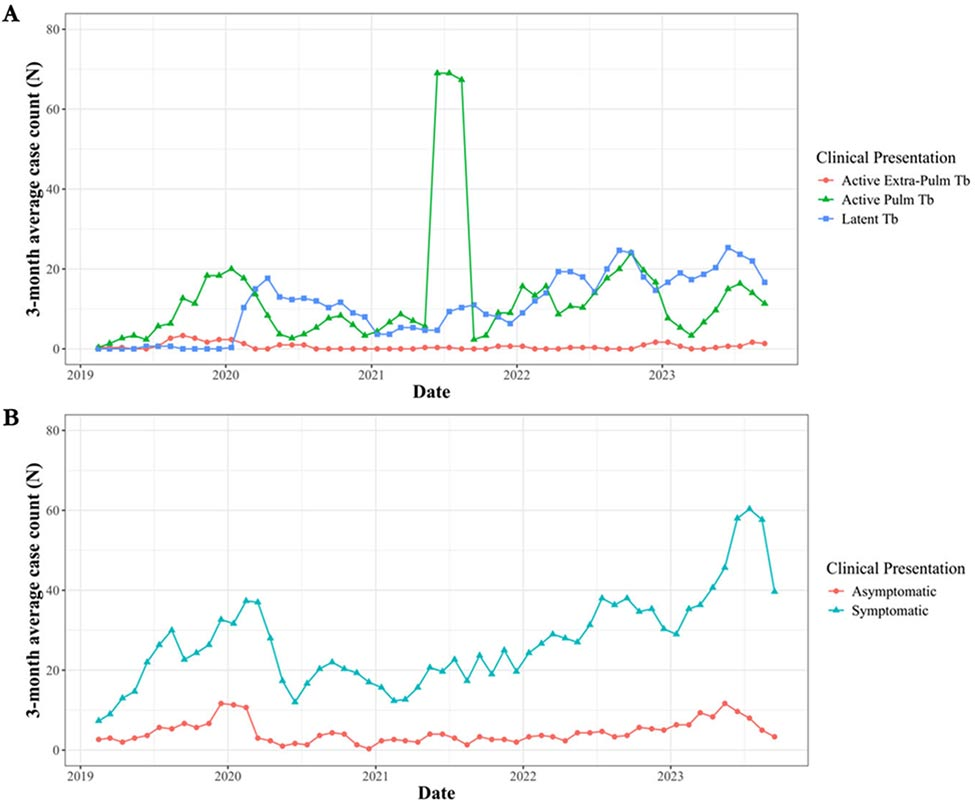

Panel A. 3-month average of diagnosed cases of Tuberculosis by clinical presentation; Panel B. 3-month average of diagnosed cases of HIV by clinical presentation.

Three month sliding average of tuberculosis case rate (per 100,000 person-months) by clinical presentation over time on linear and log scale stratified by reporting detention facility from 2019 through 2023.

**Figure:**
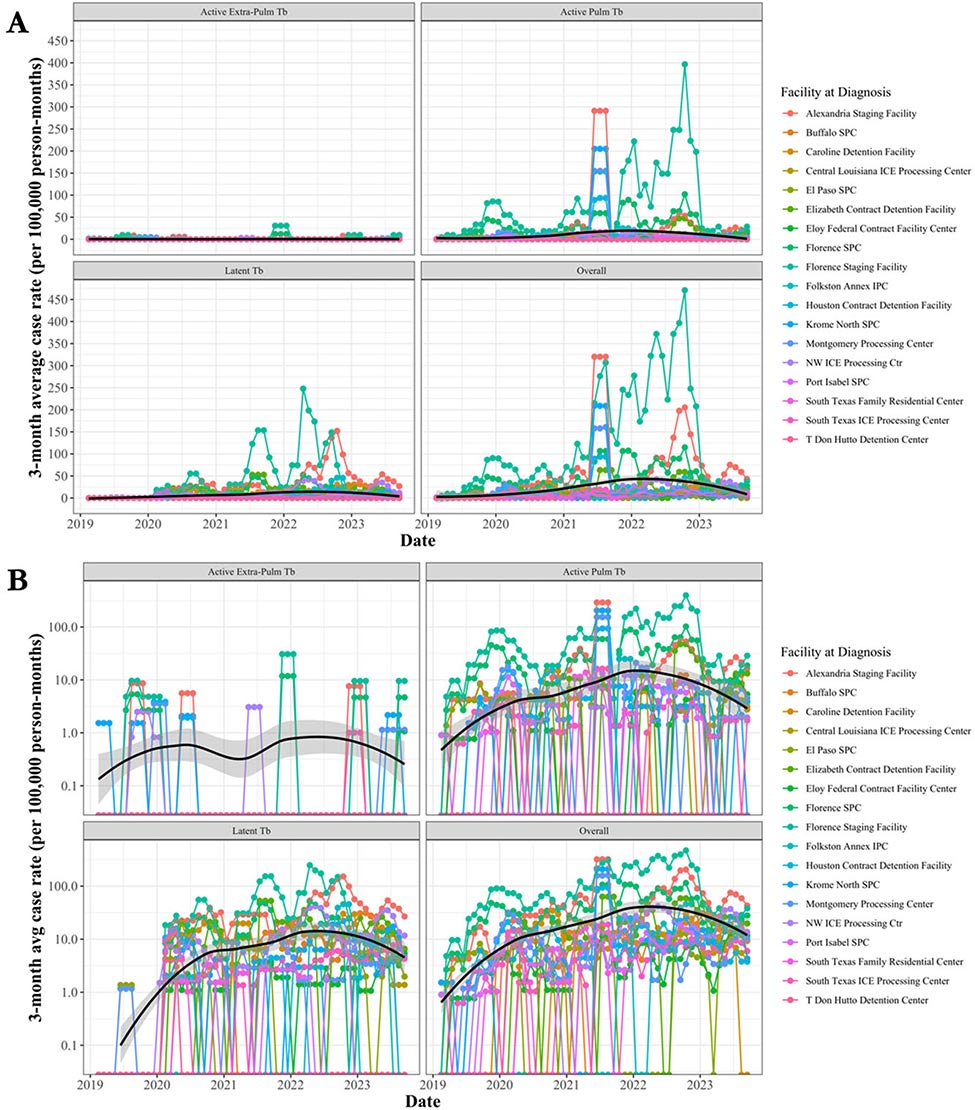

Panel A. 3-month average of case rate of Tuberculosis by clinical presentation on linear scale; Panel B. 3-month average of case rate of Tuberculosis by clinical presentation on log scale.

**Methods:**

Data on TB and HIV cases detected in ICE detention from January 2019-October 2023 were obtained from the Department of Homeland Security for 20 facilities. Cases were classified per ICD codes and aggregated monthly. Case rates (per 100,000 people-months) were estimated using annualized average daily populations (N=18).

We analyzed demographics, clinical presentation, system- and facility-level trends, and spatiotemporal variation.

Three month sliding average of HIV case rate (per 100,000 person-months) by clinical presentation over time on linear and log scale stratified by reporting detention facility from 2019 through 2023.

**Figure:**
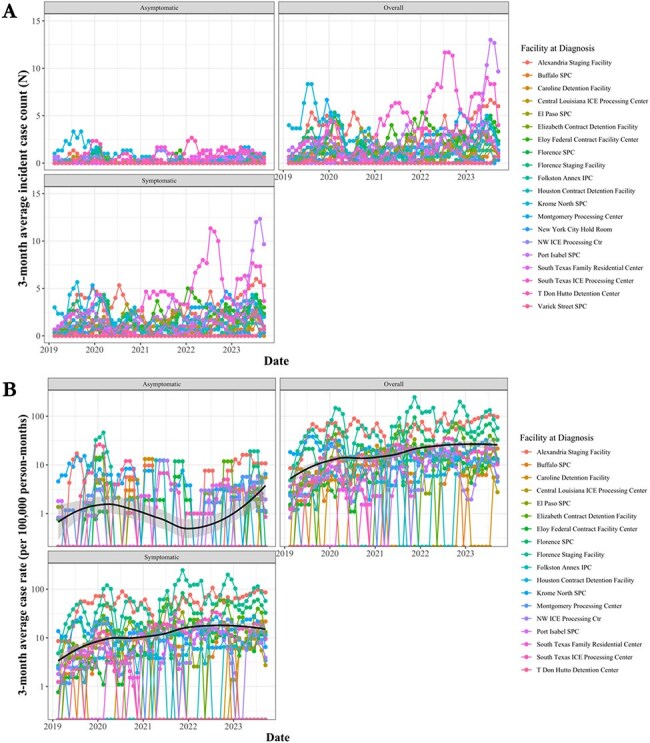

Panel A. 3-month average of case rate of HIV by clinical presentation on linear scale; Panel B. 3-month average of case rate of HIV by clinical presentation on log scale.

**Figure:**
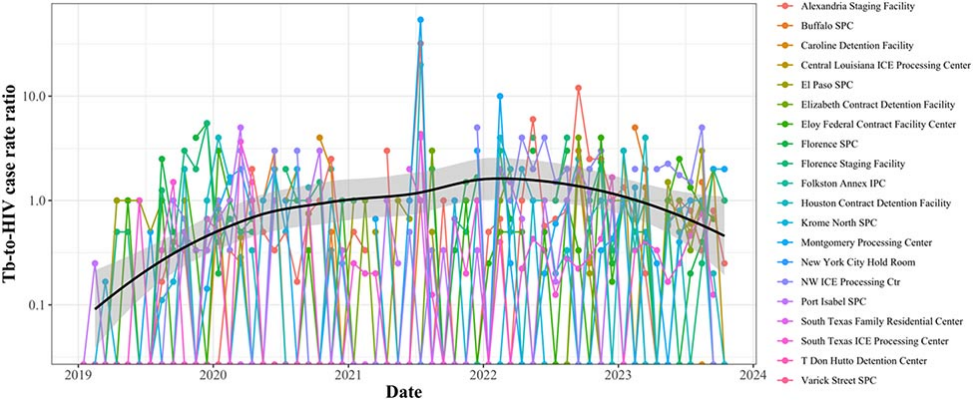
Tuberculosis to HIV case rate (per 100,000 person-month) ratio by facility from 2019 through 2023 on log scale.

**Results:**

TB: 1379 cumulative cases, averaging 23.8 monthly cases (range: 0-208) with 52.9% active pulmonary TB, 0.6% active extra-pulmonary TB, and 44.5% latent TB. The average monthly facility case rate was 9.4 (range: 0-873.3) for active pulmonary TB, 0.4 (range: 0-92.1) for active extra-pulmonary TB, and 7.2 (range: 0-520.6) for latent TB. A spatial analysis showed no autocorrelation (p=0.59). The mean coefficient of variation ranged from 2.5-5.1, indicating high monthly case rate variation.

HIV: 1802 cumulative cases, averaging 31.1 monthly cases (range: 8-78) with 85.9% symptomatic and 14.1% asymptomatic. The average monthly facility case rate was 12.8 (range: 0-297.5) for symptomatic HIV and 1.5 (range: 0-83.0) for asymptomatic HIV. A spatial analysis showed no autocorrelation (p=0.69). The mean coefficient of variation ranged from 1.3-3.7, indicating moderate monthly case rate variation. The average monthly facility TB-to-HIV case rate ratio was 0.7 (range: 0-54) and held stable over the study.

**Conclusion:**

Cases of TB and HIV, many likely endemic, remain a concern among detained migrants, with rising active pulmonary TB and symptomatic HIV case rates.

While no spatial patterns were noted, variation in case rates over time for HIV and TB suggest a review of intake protocols, reporting and diagnostic consistency and study of migrant health profiles.

Given the persistent caseload of TB and HIV, ICE has a unique opportunity for screening and sentinel surveillance to protect the health of migrants, the public and US biosecurity.

**Disclosures:**

All Authors: No reported disclosures

